# Quality of national cancer control programmes in Europe: Marjetka Jelenc

**DOI:** 10.1093/eurpub/ckac131.529

**Published:** 2022-10-25

**Authors:** M Jelenc, T Albreht

**Affiliations:** 1 Center for Health Care, National Institute of Public Health, Ljubljana, Slovenia; 2 Faculty of Health Sciences, University of Maribor, Maribor, Slovenia; 3 Medical Faculty, University of Ljubljana, Ljubljana, Slovenia

## Abstract

**Background:**

Cancer remains among the leading causes of death worldwide and the COVID-19 pandemic had a negative impact on its diagnosis and treatment. National Cancer Control Programmes (NCCPs) are key documents in the field of cancer control. As they are directed at reducing cancer morbidity, mortality, and improvement of the quality of life of cancer patients, they can make a significant contribution in tackling the challenges posed by cancer in Europe. The aim of the present study, which is unique of its kind, was to evaluate the situation regarding the presence of NCCPs in Europe in 2016, as well as to analyse the presence and implementation status of the key elements of NCCPs that European Guide for Quality National Cancer Control Programmes (Guide) recommends.

**Methods:**

In the frame of the Cancer Control Joint Action a policy survey was carried out through 35 countries: EU member states, Iceland, Montenegro, Norway and Turkey, focusing on inclusion of the chapters from the Guide, participation of stakeholders, preparation, implementation and dissemination.

**Results:**

Thirty countries responded and 28 out of those had a NCCP or a similar cancer document. NCCPs were mostly single documents; 9 documents were defined as programmes, 8 as plans and 6 as strategies. The terminology was mixed in 5 countries. NCCPs were managed by the Ministries of Health and communicated to the public via websites and/or press. Drafting of NCCPs was mostly done through discussions, consensus meetings and negotiations. Only 10 countries included all elements suggested in the Guide in their NCCPs.

**Conclusions:**

The results of the survey showed that there are still countries without a NCCP. Regarding the content of NCCPs and other cancer documents the results indicated that the quality of the documents should be improved by covering all key elements suggested in the Guide and adding other innovative approaches.

**Key messages:**

• Instruments for more efficient cancer management are needed; they should encompass the entire pathway of cancer control from health promotion, to survivorship and supportive care.

• The ongoing and quickly developing activities on NCCPs in Europe should be monitored regularly.

